# Supplementary material for: C-edge loops of arrestin function as a membrane anchor
Source: Nat Commun. 2017 Feb 21;8:14258. doi: 10.1038/ncomms14258 (PMC5321764; doi:10.1038/ncomms14258)
Supplement: Supplementary Information — Supplementary Figures, Supplementary Tables, Supplementary Notes, Supplementary References. [file ncomms14258-s1.pdf]

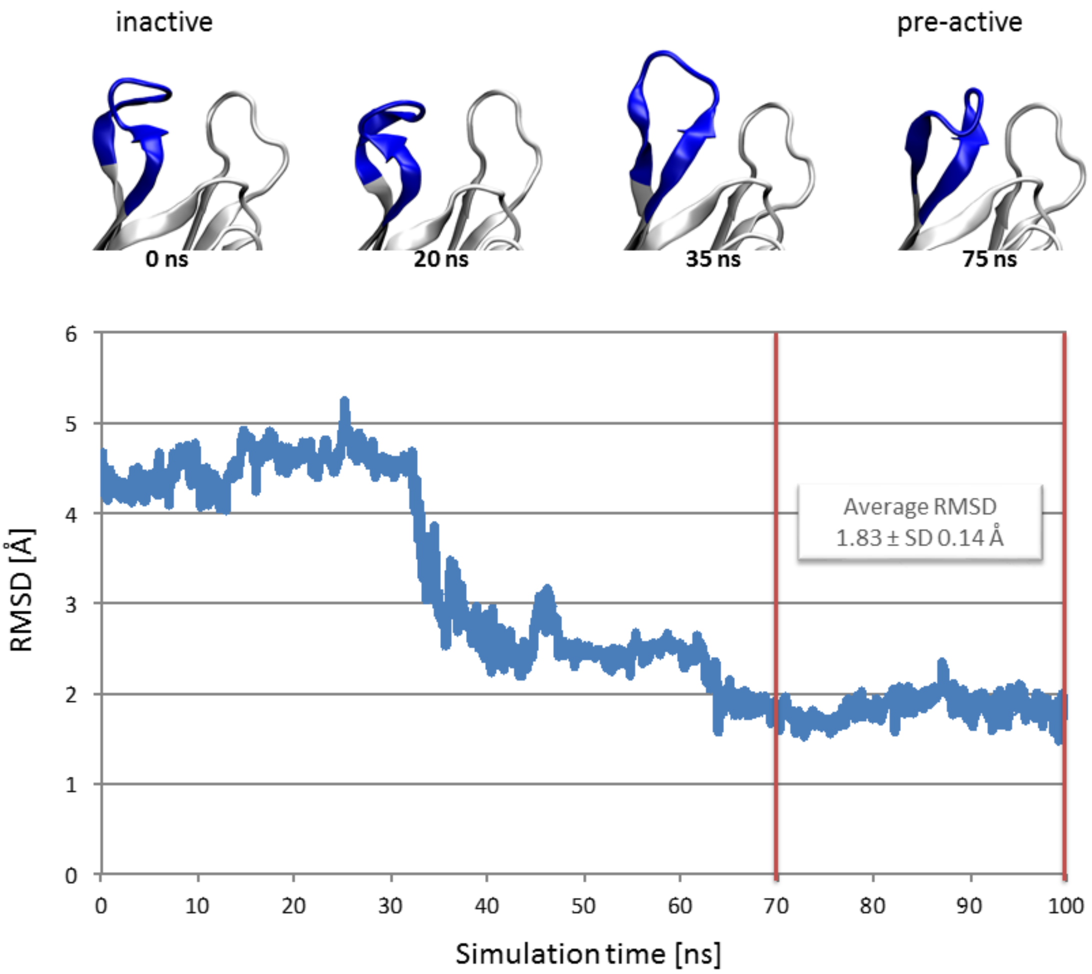

Supplementary Figure 1: Loop conversion from inactive (0 ns) to pre-active (75 ns) observed in MD2. The plot monitors the RMSD calculated for residues 335 to 345 and backbone atoms of the 344-loop using as reference the crystallized pre-active p44 structure (PDB accession code 4J2Z, chain B). After 70 ns the simulated C-terminal converges to an average RMSD of 1.83 Å.

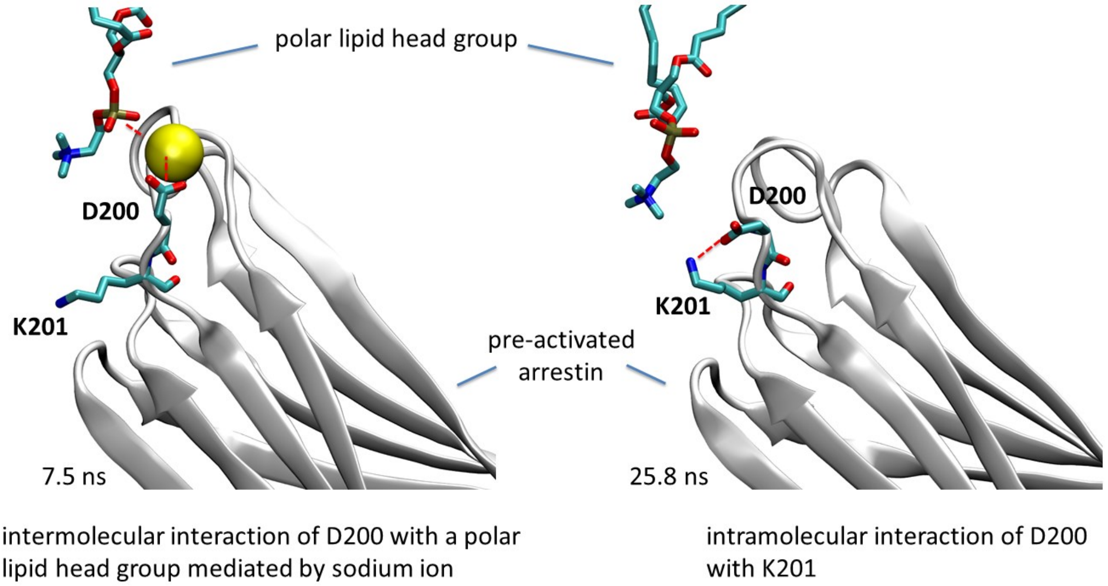

Supplementary Figure 2: Polar interactions of the arrestin C-edge with membrane phospholipids. Polar interaction between negatively charged D200 and negatively charged phosphate groups of SDPC. During the process of loop insertion into the membrane, D200 interacts with negatively charged phospholipid head groups (7.5 ns) via a positively charged sodium ion (yellow VdW) before undergoing a conformational change which allows for frequent intramolecular interaction with K201 (25.8 ns).

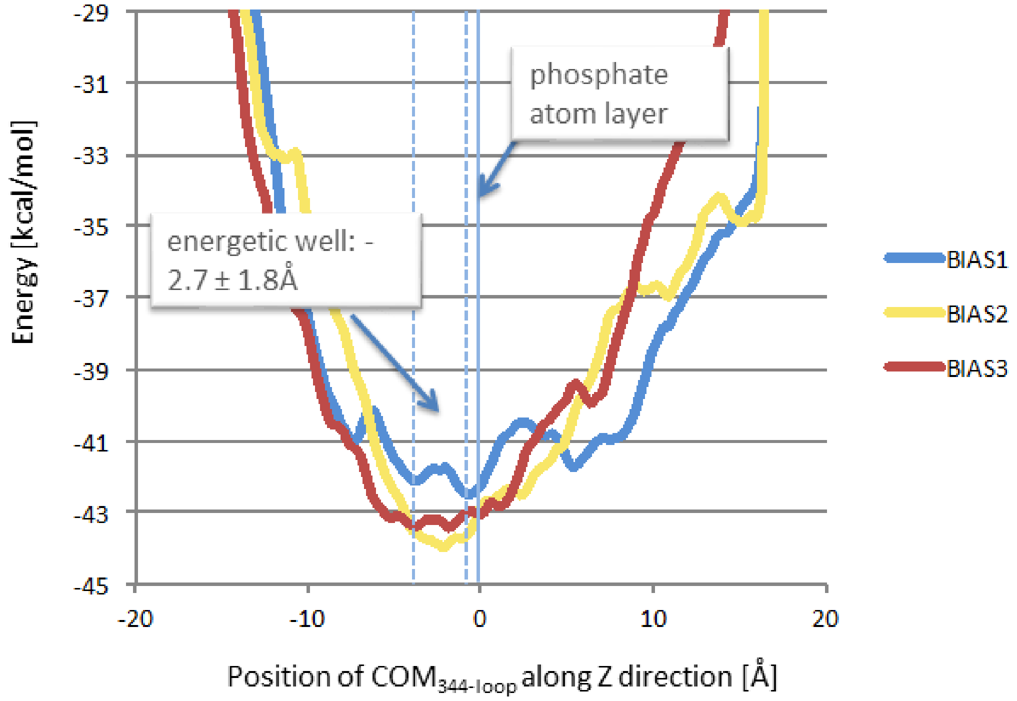

Supplementary Figure 3: Biased simulations of pre-active arrestin p44 C-edge interaction with the membrane. Metadynamics simulation of 3 replicates each 100 ns of the C-domain of the pre-activated arrestin (pdb 4J2Z, chain B). The metadynamics parameters are set to a Gaussian hill height of 2 kcal mol^{-1} with a spread of 0.2 Å for the z coordinate. The plot reveals an energetic well for the position of COM344-loop of 2.7 ± 1.8Å above the phosphate atoms.

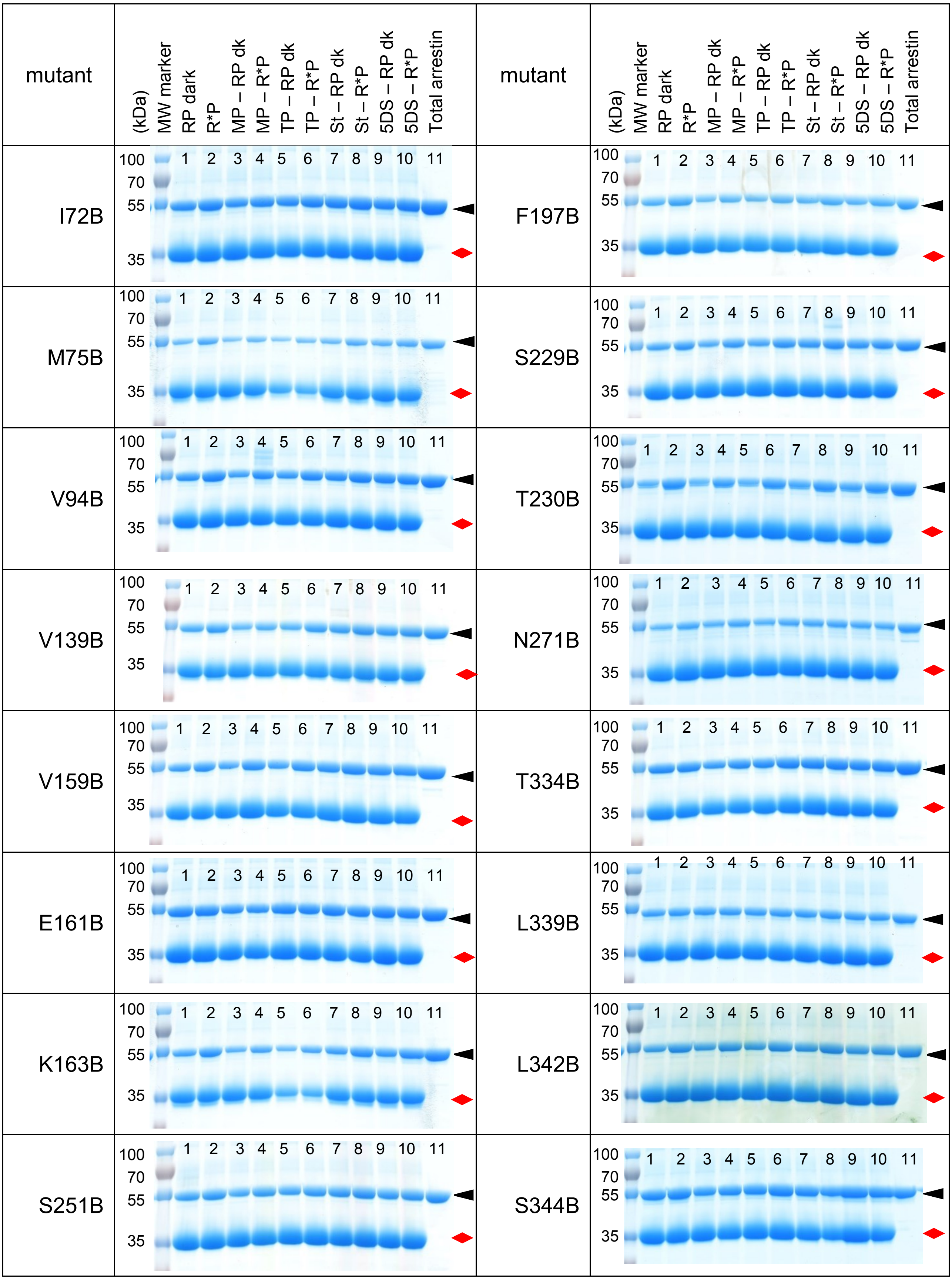

Supplementary Figure 4: Centrifugal pull-down analysis of bimane-labelled arrestin mutants. Experiments employed ROS-P membranes, which had been enriched with different fatty acids (MP, methyl palmitate; TP, N-tempoyl-palmitamide; St, stearic acid; 5DS, 5-doxyl-stearic acid). See Methods for experimental details. The black arrowheads indicate the position of arrestin on the gels, and the red diamonds indicate the position of rhodopsin. Molecular weight markers are indicated to the left of each gel.

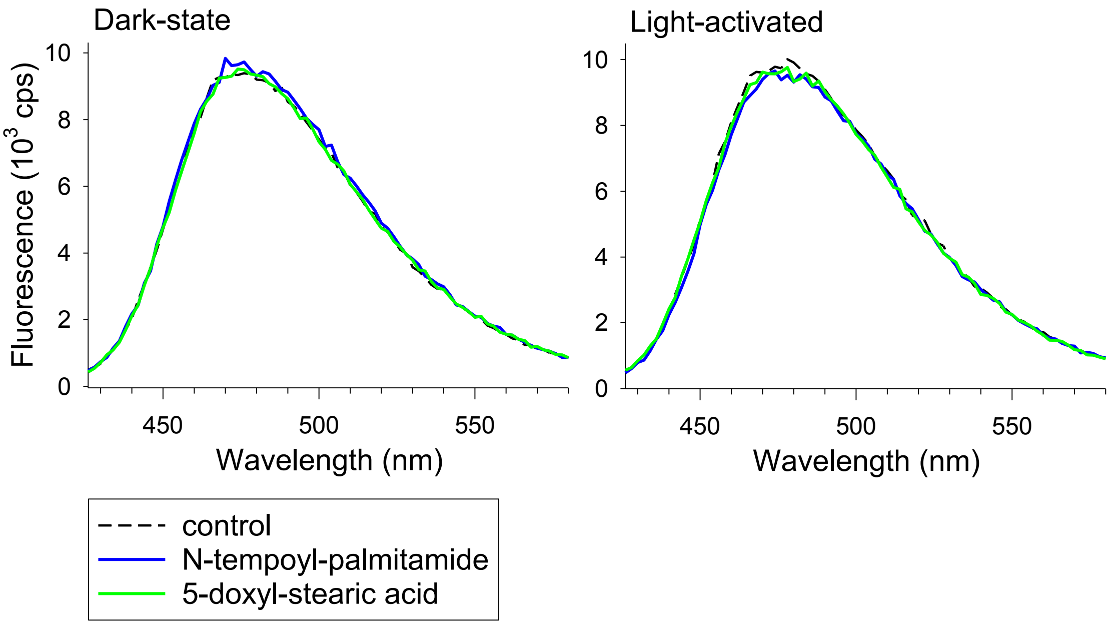

Supplementary Figure 5: Lack of interaction between arrestin and ROS membranes containing nonphosphorylated rhodopsin. The fluorescence (λex: 400 nm) of arrestin mutant S344B (1 μM) was measured in the presence of a 20-fold molar excess of nonphosphorylated rhodopsin in native ROS membranes in the dark-state (left) and after light-activation (right). No difference in fluorescence was observed between control membranes lacking spin-labelled fatty acids (black dashed spectra) and those containing N-tempoyl-palmitamide (blue spectra) or 5-doxyl-stearic acid (green spectra). Background-subtracted steady-state fluorescence spectra are shown.

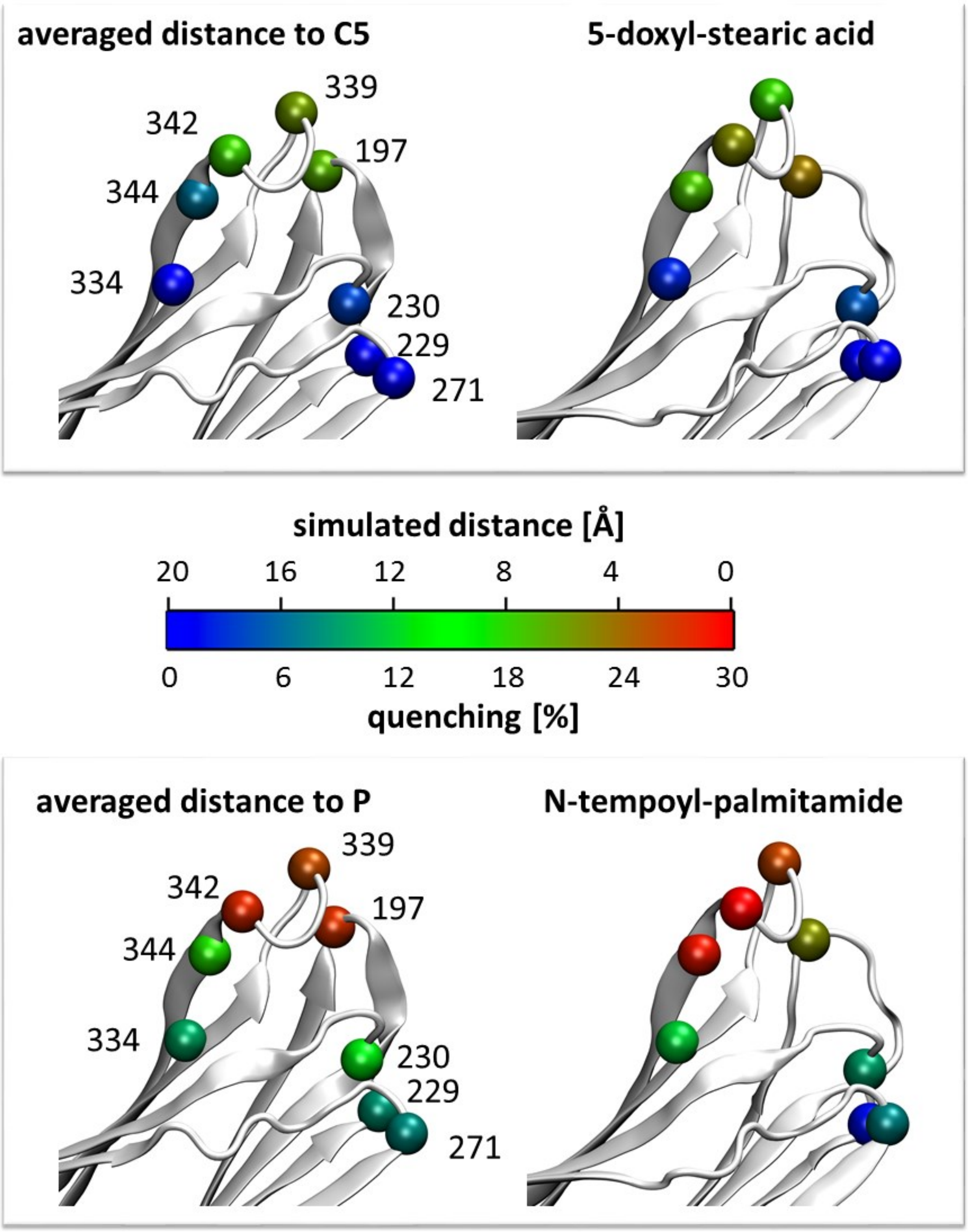

Supplementary Figure 6: Comparison of simulation and fluorescence quenching data. Left – Distances derived from all-atom simulation (MD3) averaged over the last 50 ns of simulation. The colour spectrum (0 to 20 Å) corresponds to the distance of the C-alpha carbon (VdW representation) of residues on arrestin to phosphate atoms (relates to the position of the spin label in N-tempoyl-palmitamide) and C5 atoms (relates to the position of the spin label in 5-doxyl stearic acid). Right – Quenching efficiencies derived from site-directed fluorescence experiments on the high-affinity complex are coloured according to the degree of quenching (0 to 30%). The white asterisk indicates instances of negative quenching (see main text for details).

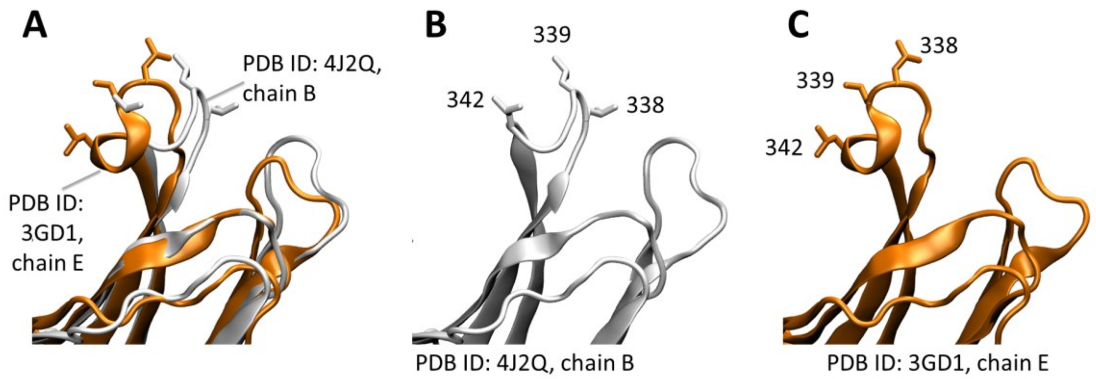

Supplementary Figure 7: Leucine orientation (Leu338, L339, L342) in the 344-loop of (**B**) pre-activated arrestin-1 (p44) (4J2Q, chain B) and (**C**) arrestin-2 bound to clathrin (PDB ID 3GD1, chain E) which are superimposed in (**A**).

Supplementary Table 1. Stability of the simulated isolated C-domain with respect to the crystallized C-domain of full-length arrestin

simulation ID	average RMSD*	standard deviation	min RMSD	max RMSD	reference structure
**MD1**	0.783	0.130	0.490	1.296	1CF1 chain D
**MD2**	1.362	0.135	0.842	1.796	1CF1 chain D
**MD3**	1.622	0.199	0.920	2.288	4J2Q chain B
**MD4**	1.602	0.191	1061	2.169	4J2Q chain B

*RMSD is computed over the backbone atoms of the entire C-domain not including loops

Supplementary Table 2. Structures of fatty acids used in this study

Methyl palmitate	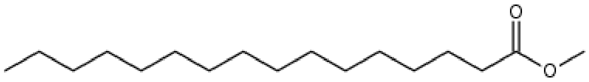
N-tempoyl palmitamide<br><br>*4-Palmitamido-2,2,6,6-tetramethylpiperidine-1-oxyl*	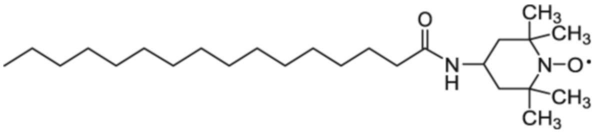
Stearic acid	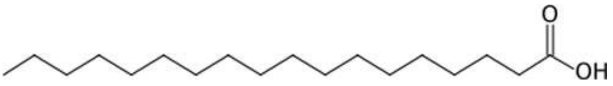
5-doxyl-stearic acid<br><br>*2-(3-Carboxypropyl)-4,4-dimethyl-2-tridecyl-3-oxazolidinyloxy*	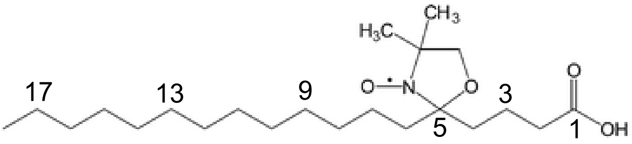

**Supplementary Table 3.** Primers used to create single cysteine mutants used in this study		
Mutation	Primers	Codon change
I72C	Fw. GCCCATCACGTCacaGTCTTCCTGGCC<br>Rev. GGCCAGGAAGACtgtGACGTGATGGGC	ATC → TGT
M75C	Fw. GCGGAAGCTGAGGCCgcaCACGTCGATGTCTTC<br>Rev. GAAGACATCGACGTGtgcGGCCTCAGCTTCCGC	ATG → TGC
V94C	Fw. GGTGGCGCCCGAGGCCCCgcaGGGAGGGAACACCTGGAC<br>Rev. GTCCAGGTGTTCCCTCCCtgcGGGGCCTCGGGCGCCACC	GTG → TGC
V139C	Fw. AGCGCTCTTGCCacaATCTTGCGGAGCCGG<br>Rev. CCGGCTCCGCAAGATtgtGGCAAGAGCGCT	GTG → TGT
V159C	Fw. AATTTTGTCCTCTTCacaATCTGTGCTGTG<br>Rev. CACAGCACAGATtgtGAAGAGGACAAAATT	GTG → TGT
E161C	Fw. GGGAATTTTGTCacaTTCCACATCTGTGCT<br>Rev. AGCACAGATGTGGAAtgtGACAAAATTCCC	GAG → TGT
K163C	Fw. CTTCTTGGGAATacaGTCCTCTTCCACATC<br>Rev. GATGTGGAAGAGGACtgtATTCCCAAGAAG	AAA → TGT
F197C	Fw. GCTTGTCCGACATgcaGAACTGAAAGGAG<br>Rev. CTCCTTTCAGTTCtgcATGTCGGACAAGC	TTC → TGC
S229C	Fw.<br>CTTCTTCACTGTCTTCTCTGTacaGTTGGTCACGGCCACGGTCA<br>C<br>Rev.<br>GTGACCGTGGCCGTGACCAACtgtACAGAGAAGACAGTGAAG<br>AAG	AGC → TGT
T230C	Fw. CTTCACTGTCTTCTCgcaGCTGTTGGTCACGGC<br>Rev. GCCGTGACCAACAGCtgcGAGAAGACAGTGAAGC	ACA → TGC
S251C	Fw. GTAATAATCACTacaATAGAGAACCACGTT<br>Rev. AACGTGGTTCTCTATtgtAGTGATTATTAC	TCC → TGT
N271C	Fw. CTTGGTCAGCGAGCTgcaTGGCGGCACTTTTTCC<br>Rev. GAAAAAGTGCCGCCAtgcAGCTCGCTGACCAAG	AAC → TGC
T334C	Fw. CCAGAAGGCCTGACACgcaGAGCTTCACCTTGATC<br>Rev. GATCAAGGTGAAGCTCtgcGTGTCAGGCCTTCTGG	ACG → TGC
L339C	Fw. GGATGTGAGCTCTCCacaAAGGCCTGACACCG<br>Rev. CGGTGTCAGGCCTTtgtGGAGAGCTCACATCC	CTG → TGT
L342C	Fw. GCCCATCACGTCacaGTCTTCCTGGCC<br>Rev. GGCCAGGAAGACtgtGACGTGATGGGC	CTC → TGT
S344C	Fw. AGTGGCCACTTCACTacaTGTGAGCTCTCC<br>Rev. GGAGAGCTCACAtgtAGTGAAGTGGCCACT	TCC → TGT

Supplementary Note 1. The physical mechanism of fluorophore quenching by spin label is currently not completely understood, and multiple mechanisms may be simultaneously involved. The spin label is paramagnetic and hence can be expected to quench by a similar mechanism as oxygen, which is believed to occur by enhanced intersystem crossing[1]. In the normal case, fluorescence occurs when a ground-state electron of the fluorophore absorbs a photon of the exciting light and goes to a higher orbital level. Energy is lost due to vibrational relaxation, such that when the electron returns to ground state, the absorbed energy is released as light of a longer wavelength (*i.e.* lower energy). In the presence of a paramagnetic species, the chance of intersystem crossing is increased. The excited singlet state becomes a triplet state, which is lower in energy and longer-lived, and therefore more likely to return to the ground state in a non-radiative way. Besides intersystem crossing, electron transfer is also believed to play a role in quenching by nitroxide spin labels[2,3]. In brief, the excited electron of the fluorophore is transferred to the nitroxide spin label, which transfers a ground-state electron back to the fluorophore. The spin label is left in an excited state, but quenching occurs as there is no direct transition from the excited state to the ground state of the fluorophore. .

Supplementary Note 2. In order to maximize quenching efficiency, we designed our experiments to favour complete arrestin association with phosphorylated ROS membranes in both the dark-state and following light-activation. The key considerations were a very high receptor phosphorylation level, minimal salt concentration, and an excess of receptor over arrestin. We have developed a rhodopsin phosphorylation procedure that yields preparations in which every receptor is capable of binding arrestin as light-activated Meta II and which have high affinity for arrestin even in the ligand-free state (phosphorylated opsin)[4]. Arrestin association with dark-state phosphorylated rhodopsin (Rho-P) is primarily governed by electrostatic interactions and hence is favoured by low ionic strength[4,5].

These conditions resulted in comparable arrestin binding to Rho-P and light-activated Rho*-P (see Supplementary Fig. S4), despite the very different affinities of arrestin to these two receptor species. Arrestin-1 binding affinity (K_D) for Rho-*P has been reported to be in the nanomolar range[6,7]. For dark-state Rho-P, Zhuang *et al.* reported a K_D of ~80 μM in the presence of 100 mM NaCl. However, measurements by our group in the absence of salt, and with high receptor phosphorylation, yielded a K_D of ~1 μM for arrestin binding to dark-state Rho-P[4].

Supplementary Note 3. Tryptophan (Trp) and tyrosine (Tyr) residues can quench the fluorescence of nearby bimane fluorophores[8,9]. Although native rhodopsin contains many Trp and Tyr residues, we do not believe these had an influence on the quenching of arrestin-attached bimanes observed in our experiments. Firstly, the cytoplasmic face of rhodopsin is fairly lacking Trp and Tyr residues. The closest candidates are Y60, Y74, and Y136 (bovine rhodopsin numbering), and these are located

many angstroms away from the cytoplasmic face of the helical bundle. Inspection of the Ops*–arrestin-1 fusion complex crystal structure shows that no Trp or Tyr residues on the receptor are in close proximity to sites on arrestin to which we attached bimane fluorophores. For example, Y136 on opsin is nearly 40 Å (Cα-Cα distance) away from site 344 on the C-edge of arrestin. Secondly, our experiments were designed such that if there was a quenching effect of receptor Trp or Tyr residues on bimanes on arrestin, we would have observed this in control measurements using ROS membranes that contained no spin label. These membranes were instead enriched in unlabelled fatty acids. In determining the quenching effect of spin labels, we compared the fluorescence in the presence of the spin labelled fatty acids to the fluorescence in the presence of unlabelled fatty acids. Any quenching by receptor Trp or Tyr residues would be the same between these two measurements and hence would not influence the observed quenching by spin label.

Supplementary Note 4. All arrestin mutants were created within a plasmid construct, called pMS2 within our laboratory, which consists of the bovine arrestin-1 cDNA cloned into the pET15b vector. The cDNA originates from the laboratory of V.V. Gurevich and contains a glycine insertion at residue 2. In addition, three native cysteine residues and one tryptophan residue were previously replaced by our group, yielding the mutations C63A, C128S, C143A, and W194F. The pET15b vector was developed many years ago by Novagen and is still commercially available from Merck Millipore. In the pMS2 construct, the arrestin gene is cloned into the Nco I and Xho 1 restriction sites. For sequencing, we use the T7 promoter and T7 reverse primers, which are commercially available.

The gene sequence of our basic construct lacking cysteine and tryptophan residues is (mutations to the native cDNA are underlined):

5’
ATG**GGA**AAGGCCAATAAGCCGGCGCCAAACCACGTGATCTTTAAAAAGATCTCCCGTGATAAATCGGTG
ACCATCTACCTGGGGAAGAGAGATTACATAGACCACGTTGAACGAGTAGAGCCTGTGGATGGCGTCGTGC
TGGTGGATCCTGAGCTCGTGAAGGGCAAGAGAGTGTACGTGTCTCTGACG**GCC**GCGTTCCGCTACGGCCA
GGAAGACATCGACGTGATGGGCCTCAGCTTCCGCAGGGACCTCTACTTCTCCCAGGTCCAGGTGTTCCCTC
CCGTGGGGGCCTCGGGCGCCACCACGAGGCTGCAGGAGAGCCTCATCAAGAAGCTGGGGGCCAACACCT
ACCCCTTCCTGCTCACGTTTCCTGACTACTTGCCC**TCC**TCGGTGATGCTGCAGCCGGCTCCGCAAGATGTG
GGCAAGAGC**GCT**GGGGTCGACTTTGAGATCAAAGCATTCGCCACGCACAGCACAGATGTGGAAGAGGAC
AAAATTCCCAAGAAGAGCTCCGTGCGTCTGCTGATCAGGAAGGTACAGCATGCGCCGCGGGATATGGGTC
CCCAGCCCCGAGCCGAGGCCTCC**TTT**CAGTTCTTCATGTCGGACAAGCCCCTGCGCCTCGCCGTCTCGCTC
AGCAAAGAGATCTATTACCACGGGGAACCCATTCCTGTGACCGTGGCCGTGACCAACAGCACAGAGAAG
ACAGTGAAGAAGATTAAAGTGCTAGTGGAGCAAGTGACCAACGTGGTTCTCTATTCCAGTGATTATTACA
TCAAGACGGTGGCTGCGGAGGAAGCACAGGAAAAAGTGCCGCCAAACAGCTCGCTGACCAAGACGCTGA
CGCTGGTGCCCTTGCTGGCCAACAACCGTGAGAGAAGGGGCATCGCCCTGGATGGGAAAATCAAGCACG
AGGACACGAACCTGGCCTCCAGCACCATCATAAAGGAGGGAATAGACAAGACCGTCATGGGGATCCTGG
TGTCTTACCAGATCAAGGTGAAGCTCACGGTGTCAGGCCTTCTGGGAGAGCTCACATCCAGTGAAGTGGC
CACTGAGGTGCCGTTCCGCCTCATGCATCCCCAGCCAGAGGACCCAGATACCGCCAAGGAAAGTTTTCAG
GATGAAAATTTTGTTTTTGAGGAGTTTGCTCGCCAAAATCTGAAAGATGCAGGAGAATATAAGGAAGAGA
AGACAGACCAGGAGGCGGCTATGGATGAGtga 3’

The corresponding protein sequence is:

M**G**KANKPAPNHVIFKKISRDKSVTIYLGKRDYIDHVERVEPVDGVVLVDPELVKGKRVYV
SLT**A**AFRYGQEDIDVMGLSFRRDLYFSQVQVFPPVGASGATTRLQESLIKKLGANTYPFLLT
FPDYLP**S**SVMLQPAPQDVGKS**A**GVDFEIKAFATHSTDVEEDKIPKKSSVRLLIRKVQHAPRD
MGPQPRAEAS**F**QFFMSDKPLRLAVSLSKEIYYHGEPIPVTVAVTNSTEKTVKKIKVLVEQV
TNVVLYSSDYYIKTVAAEEAQEKVPPNSSLTKTLTLVPLLANNRERRGIALDGKIKHEDTN
LASSTIIKEGIDKTVMGILVSYQIKVKLTVSGLLGELTSSEVATEVPFRLMHPQPEDPDTAKE
SFQDENFVFEEFARQNLKDAGEYKEEKTDQEAAMDE*

Supplementary References

1 Lakowicz, J. R. *Principles of Fluorescence Spectroscopy*. 2nd edn, Vol. 3rd ed. (Kluwer Academic, 1999).

2 Fayed, T. A., Grampp, G. & Landgraf, S. Fluorescence quenching of aromatic hydrocarbons by nitroxide radicals: a mechanismatic study. *Int J Photoenergy* **1** (1999).

3 Matko, J., Ohki, K. & Edidin, M. Luminescence quenching by nitroxide spin labels in aqueous solution: studies on the mechanism of quenching. *Biochemistry* **31**, 703-711 (1992).

4 Sommer, M. E., Hofmann, K. P. & Heck, M. Distinct loops in arrestin differentially regulate ligand binding within the GPCR opsin. *Nat Commun* **3**, 995, doi:10.1038/ncomms2000 (2012).

5 Gurevich, V. V. & Benovic, J. L. Visual arrestin interaction with rhodopsin. Sequential multisite binding ensures strict selectivity toward light-activated phosphorylated rhodopsin. *J Biol Chem* **268**, 11628-11638 (1993).

6 Pulvermüller, A. *et al.* Functional differences in the interaction of arrestin and its splice variant, p44, with rhodopsin. *Biochemistry* **36**, 9253-9260, doi:10.1021/bi970772g (1997).

7 Bayburt, T. H. *et al.* Monomeric rhodopsin is sufficient for normal rhodopsin kinase (GRK1) phosphorylation and arrestin-1 binding. *J Biol Chem* **286**, 1420-1428 (2011).

8 Jones Brunette, A. M. & Farrens, D. L. Distance Mapping in Proteins Using Fluorescence Spectroscopy: Tyrosine, like Tryptophan, Quenches Bimane Fluorescence in a Distance-Dependent Manner. *Biochemistry* **53**, 6290-6301, doi:10.1021/bi500493r (2014).

9 Mansoor, S. E., Dewitt, M. A. & Farrens, D. L. Distance mapping in proteins using fluorescence spectroscopy: the tryptophan-induced quenching (TrIQ) method. *Biochemistry* **49**, 9722-9731, doi:10.1021/bi100907m (2010).
